# Beneficial effects of a novel inspiratory muscle training device on respiratory muscle strength and submaximal functional capacity in community-dwelling older Thai adults: a randomized controlled trial

**DOI:** 10.7717/peerj.21072

**Published:** 2026-04-07

**Authors:** Naruemon Garnkhan, Chusak Thanawattano, Kornanong Yuenyongchaiwat

**Affiliations:** 1 Department of Physiotherapy, Faculty of Allied Health Sciences, Thammasat University, Pathumthani, Thailand; 2 National Institute of Development Administration, Pathumthani, Thailand; 3 Thammasat University Research Unit for Physical Therapy in Respiratory and Cardiovascular Systems, Thammasat University, Pathumthani, Thailand

**Keywords:** Inspiratory muscle training, Breathing exercise, Respiratory muscle, Older adults, Submaximal functional capacity

## Abstract

Older adults encounter progressive deterioration of all organ systems. Disease development reportedly increases with age, resulting in decreased physical performance in terms of aerobic endurance, muscular strength, and physical ability. Inspiratory muscle training (IMT) is commonly performed to improve cardiorespiratory fitness. Therefore, this study aimed to develop a resistive inspiratory muscle and determine the effect of inspiratory muscle training on respiratory muscle strength and functional capacity in older adults.

**Methods:**

Sixty older adults (age: ≥60 years), both males and females, were enrolled. All participants performed a respiratory muscle strength test (maximal inspiratory pressure (MIP), maximal expiratory pressure (MEP)) and a 6-minute walking distance test (*i.e.*, submaximal functional capacity) before and after an 8-week intervention program. An inspiratory muscle training device, the Breath Trainer, an air-resistive respiratory training device connected to a smartphone via Bluetooth, was developed. Each individual was required to attach the Breath Trainer to the lower costal part of the chest wall during the training program. The breathing training group used a Breath Trainer set to 40% MIP, whereas the sham-IMT performed a training set to 0% MIP. In both groups, training involved 15 repetitions, three sessions/day, and 5 days/week. An intention-to-treat analysis was performed, followed by a two-way mixed repeated analysis of variance (ANOVA).

**Results:**

Between-group difference, the breathing training group exhibited improved respiratory muscle strength (MIP = 19.56 ± 4.54 cmH_2_O, *p* < .001, MEP = 18.87 ± 43.41 cmH_2_O, *p* < .001) and submaximal functional capacity (56.63 ± 17.97 meters, *p* = .003) compared with the sham-IMT, following the 8-week program.

**Discussion:**

An 8-week program with the Breath Trainer could improve respiratory muscle strength and submaximal functional capacity in older adults.

## Introduction

Aging is associated with reduced skeletal muscle strength, which, in turn, affects the respiratory muscles. From the fourth decade of life, maximal inspiratory pressure (MIP) and maximal expiratory pressure (MEP) reportedly decline by ∼1% annually due to changes in respiratory structure and movement of the external structures around the lungs, along with a reduction in inhalation and exhalation (Harik-Khan, Wise & Fozard, 1998). Factors affecting chest movement include age, sex, and posture, which affect lung volume (Kaneko & Horie, 2012; Mendes et al., 2020). Age is associated with loss of muscle mass (sarcopenia) and functional decline in skeletal muscles, including the main inspiratory muscle, which, in turn, reduces the functional performance of the diaphragm muscle. Therefore, increased respiratory effort is typically associated with increased dyspnea and decreased physical activity. During aging, the transdiaphragmatic pressure decreases by 20–41%, while respiratory muscle strength is reduced by 30% (Bordoni, Morabito & Simonelli, 2020). These changes in the diaphragm may contribute to aging-related respiratory complications. Furthermore, older adults frequently exhibit reduced exercise tolerance and lower physical performance, which can be linked to poor muscle mass and reduced cardiovascular capacity and joint mobility (American College of Sports Medicine et al., 2009). Additionally, lower respiratory muscle strength has been associated with reduced physical function owing to its effects on respiratory function (Dourado et al., 2006; Watsford, Murphy & Pine, 2007; Giua et al., 2014). In the respiratory system, most aging-related changes evolve from a decrease in chest wall compliance, a reduction in static elastic recoil of the lungs, and a decrease in respiratory muscle strength due to dyspnea, which is a major symptom that leads to fatigue and reduced physical activity, resulting in muscle weakness, heart malfunction, reduced physical activity, malnutrition, anxiety, and depression, which are major concerns impacting the quality of life (Lalley, 2013; Stewart & King, 1991).

In older adults, inspiratory muscle training (IMT) was found to enhance respiratory dynamics and functional exercise capacity, possibly improving the overall quality of life (Aznar-Lain et al., 2007). Souza et al. (2014) evaluated the efficacy of IMT on respiratory strength, diaphragm thickness, and diaphragmatic mobility in older women and found that 8 weeks of IMT at 40% MIP improved respiratory muscle strength, diaphragm thickness, and mobility in the participants. In patients with atrial fibrillation, Zeren et al. (2016) examined the effects of IMT on pulmonary function, respiratory muscle strength, and functional capacity at 30% MIP, 15 min twice daily, 7 days/week, for 12 weeks to improve pulmonary function, respiratory muscle strength, and functional capacity. Furthermore, Shin et al. (2017) evaluated the sarcopenic indices of respiratory muscle strength in older adults and suggested that respiratory muscles, especially inspiratory muscles, are substantially associated with limb muscle strength and skeletal muscle mass.

Accumulated evidence suggests that IMT is commonly performed to improve respiratory muscle strength. Therefore, we developed a device to perform IMT *via* a smartphone application using a machine designed to enhance breathing muscle strength. This device can simultaneously measure heart rate and respiratory muscle strength, displaying the results as numerical values and graphs on the smartphone screen (Yuenyongchaiwat et al., 2019; Yuenyongchaiwat et al., 2021). In addition, the commercial respiratory-training devices are typically used by mouth, whereas the Breath Trainer is applied directly to the lower costal region. This allows the respiratory muscles, such as the diaphragm and intercostal muscles, to be specifically targeted, whereas mouth-used devices, such as POWERbreathe, may also engage accessory muscles. Accordingly, we aimed to determine the effects of a breathing training device on respiratory muscle strength, submaximal functional capacity, and physical performance in older adults.

## Materials & Methods

Ethical approval has been received from the Human Research Ethics Committee of Thammasat University (Science), Thailand. (ethics approval number: COA No. 066/2564). Each participant received an information sheet and signed a written informed consent form prior to participation. The study was registered on the Thai Clinical Trials RegistryTCTR20211006002.

This randomized controlled trial involved 60 older adults aged ≥60 years and recruited male and female participants. The sample size was calculated with the G-power program 3.1.9.4, and the effect size f was set at 0.25 (medium effect size) based on IMT literature, which has generally reported small-to-medium improvements in MIP and related outcomes (Seixas et al., 2020; Mills et al., 2015; Manifield et al., 2021). Reported effect sizes range from approximately *d* = 0.35–0.70 (equivalent to f ≈ 0.18–0.35). Therefore, *f* = 0.25 was selected as a conservative and appropriate estimate. A power level of 0.95 was used to reduce the risk of type II error and ensure adequate sensitivity to detect potential changes. Therefore, 54 individuals were calculated. However, to prevent missing data or dropout, the study enrolled 60 participants.

All participants were recruited from community dwellings. Participants were eligible if they had no cognitive impairment (assessed by The Montreal Cognitive Assessment: MoCA ≥ 26) and were physically inactive. Physical activity level was assessed using the Global Physical Activity Questionnaire (GPAQ), following the scoring protocol recommended by the World Health Organization (WHO). The GPAQ quantifies physical activity across work, transport, and recreational domains. Total physical activity (metabolic equivalent (MET)-min/week) was calculated by multiplying minutes per week of moderate-intensity activity by 4 METs and vigorous-intensity activity by 8 METs, then summing across all domains. Participants with total physical activity <600 MET-min/week—classified by the WHO as physically inactive (Bull, Maslin & Armstrong, 2009). Therefore, older people with low physical activity (GPAQ < 600 MET-min/week) were eligible for inclusion in the study.

**Figure 1:**
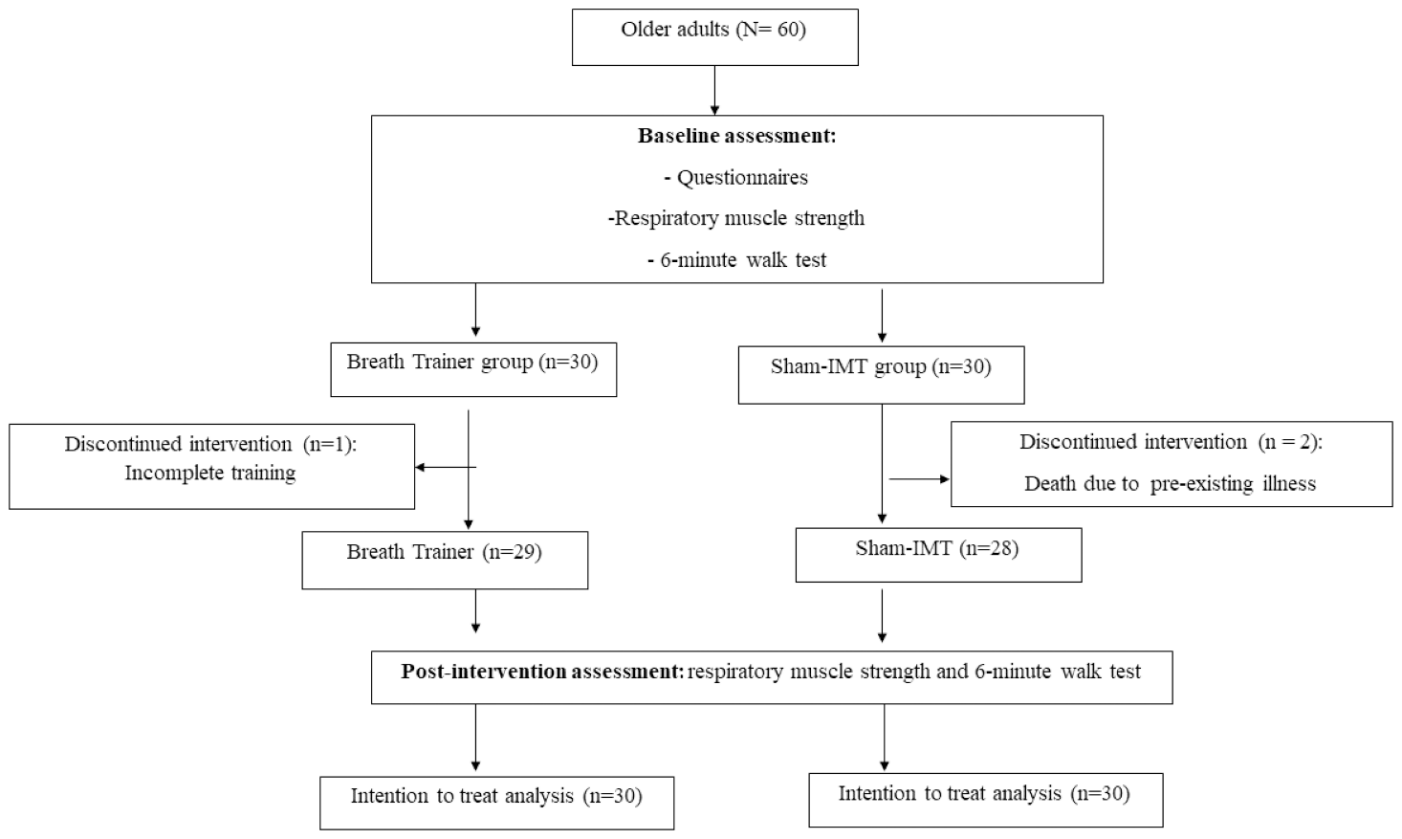
Flow chart. Recruitment of participants.

Using a simple random sampling technique, individuals were randomly assigned to breathing training and sham-IMT. The intervention comprised eight weeks of combined training, with measurements before and after completion. The participants used a respiratory pressure meter and performed a 6-minute walk test (6-MWT) before and after the 8-week intervention program. All baseline and post-intervention assessments were conducted at a community center to ensure convenient access for participants and to facilitate their ability to complete the required tests. The inspiratory muscle training and sham training sessions were performed at home, supported by the device’s smartphone application. The participants were randomized into two groups (breathing training and sham-IMT). The study was conducted as an assessor-blind randomized controlled trial. Due to the nature of the intervention and the use of a 0% MIP sham condition, full blinding of participants was not feasible. Data analysis was performed by an investigator who was not involved in intervention delivery and was blinded to group assignment. A lottery method is used for a simple randomized sampling technique. Group assignments were prepared in advance on identical slips of paper, sealed inside opaque envelopes, and shuffled. An independent research assistant who was not involved in participant recruitment or outcome assessment opened the envelopes after baseline testing to determine group allocation. This procedure ensured allocation concealment and prevented investigators from predicting or influencing group assignment. The study procedure followed the CONSORT flow diagram (Fig. 1).

**Figure 2:**
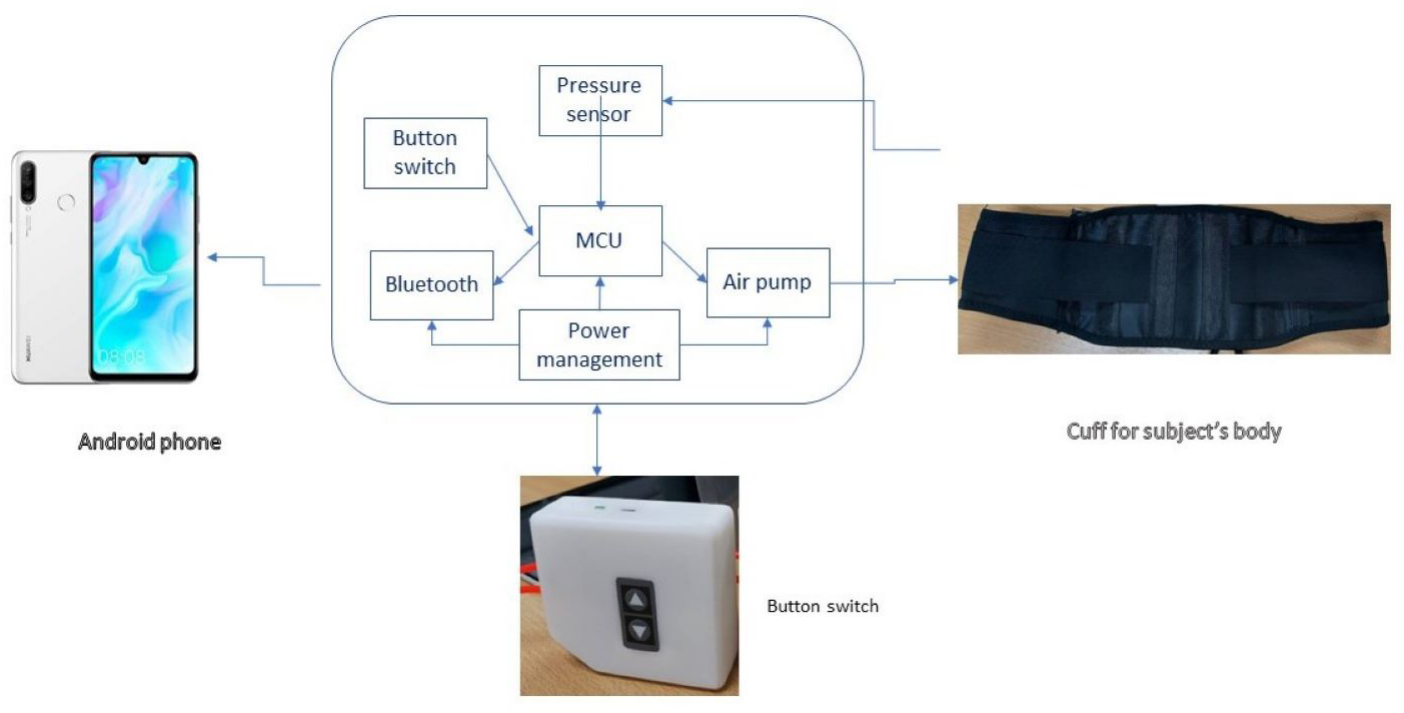
Breath Trainer composition.

The breathing training device, the Breath Trainer, has three main parts: a pressure sensor, a lower-costal cuff with an air pump, and a smartphone with a dedicated application (Fig. 2). The training system comprises two main parts: an air-resistive respiratory training device and a smartphone with a dedicated application. In the air-resistive training device, the air cuff was covered with fabric tailored to fit a normal-sized adult waist and filled with air using an air pump. The air cuff is connected to the pressure sensor *via* a flexible rubber pipe reinforced with a cable tie to prevent air leakage at the sensor-side port. The pressure sensor used in the device is the MP3V5050GP, which measures pressure in the range 0–50 kPa (approximately 0–510 cmH_2_O, using 1 kPa ≈ 10.197 cmH_2_O) with a specified accuracy of 2.5% (NXP Semiconductors, 2018). The sensor operates at a typical supply voltage of 3.3 V and provides a near-linear output spanning approximately 0.188–2.888 V across the full pressure range, corresponding to an effective sensitivity consistent with the previously stated 54 mV/kPa.

Calibration of the pressure sensor was performed prior to data collection using a calibrated digital manometer (traceable to national standards). Known static pressures were applied at multiple points across the operating range, and a linear calibration (slope and offset) was derived to convert sensor output voltage to pressure. Calibration was re-verified by a zero-offset check before each session and repeated after any hardware modification (*e.g.*, sensor replacement or tubing/cuff changes).

The microcontroller (MCU) displayed the pressure value through an analog-to-digital module with a sampling rate of 10 Hz. Subsequently, the MCU forwarded the formatted data to the smartphone through Bluetooth communication. Finally, the breathing training data were recorded. The Breath Trainer device was tested by the Electrical and Electronic Products Testing Center of the National Science and Technology Development Agency, Thailand, for electrical safety in accordance with the International Electrotechnical Commission (IEC) 60601-1. This testing supports the device’s electrical safety for intended use. This certification confirms only electrical and operational safety and does not verify measurement accuracy. Pressure measurement performance is supported by the pressure sensor datasheet specifications and by a prior study reporting the validity of the device for measuring MIP (Yuenyongchaiwat et al., 2017). Together, these sources support the accuracy and consistency of the pressure measurements obtained during the intervention.

The breathing training group received a breathing training device set to 40% MIP. The sham intervention consisted of breathing with the device set at 0% MIP, resulting in load-free breathing. The 0% MIP setting was chosen to ensure that the control group followed a training protocol that was as closely matched as possible to the intervention group. This design allowed participants to experience the same training procedures and structure, thereby maintaining engagement and a sense of participation in the intervention. Therefore, we selected 0% MIP (unloaded breathing) as the sham condition. In addition, both groups were prescribed the same number of repetitions and training sessions, with the only difference being the level of inspiratory load applied. Resistance was applied externally using an inflatable abdominal cuff that opposed abdominal expansion during inspiration. Mechanically, the cuff increases the load on the diaphragm by restricting the normal outward displacement of the abdominal wall. As a result, inspiratory muscles must have generated greater negative intrathoracic pressure and diaphragmatic force to achieve the same tidal volume. As this mechanism differed from that used by mouth-applied pressure-threshold devices, we explicitly trained participants in diaphragmatic (abdominal) breathing prior to commencing the intervention. Training comprised a standardized instruction session (10–15 min) delivered by a physiotherapist, including demonstration, supervised practice, and corrective feedback until the participant could produce consistent abdominal excursions on three consecutive breaths. Participants then completed two supervised practice sessions at the community center to confirm the technique before home training began. The smartphone application provided reminders and logged session completion. Study staff reviewed adherence and technique-related notes weekly and refreshed coaching if inconsistent patterns were detected.

**Figure 3:**
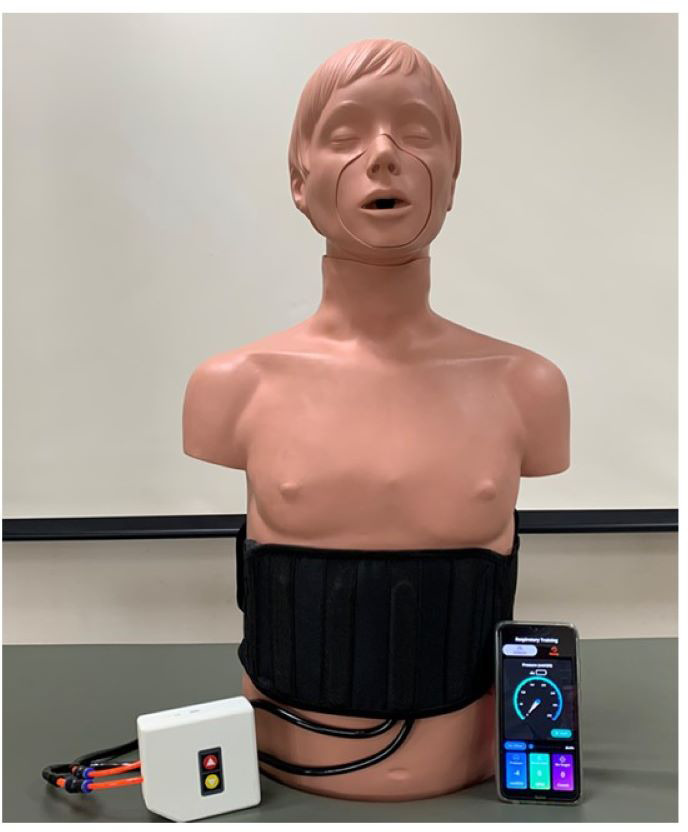
Breath Trainer device.

Training intensity was prescribed relative to MIP and set to ∼40% MIP, a level intended to provide a moderate but safe training stimulus for older adults while limiting fatigue and adverse events. The 40% target was selected to balance feasibility, tolerability, and the risk of low-intensity loads producing measurable training effects. We recognize that this represents a lower intensity than some high-load IMT protocols; the choice was guided by population safety and prior feasibility work. Both groups performed 15 repetitions, three sessions/day, and 5 days/week. Participants were required to attach the chest strap to the lower margin of the costal space (Fig. 3). During the intervention protocols, both groups returned to the laboratory after four weeks to perform the MIP and MEP assessment and adjust the training load. After 8 weeks, the participants returned to determine the post-test measurements.

Adherence to the intervention was monitored using the smartphone application linked to the training device. Due to the smartphone application’s features, the device’s detailed usage history, including session duration, training dose, and training frequency, was automatically recorded for each participant. A completed session was defined as the performance of the full prescribed number of breaths at the assigned resistance level within a training session. Sessions that did not reach the preset number of breaths were classified as incomplete. According to these records, all participants in both the IMT and sham groups completed all prescribed training sessions, resulting in 100% adherence and equivalent dose delivery across groups.

All participants were assessed for respiratory muscle strength and submaximal functional capacity at baseline (*i.e.,* before the intervention program) and 8 weeks after training completion by a physical therapist. Respiratory muscle strength was assessed using the MicroRPM^®^ Respiratory Pressure Meter (Micro Medical/CareFusion, Kent, United Kingdom) by the same trained assessor to ensure intra-rater reliability. The protocol is followed by the American Thoracic Society protocol (American Thoracic Society/European Respiratory Society, 2002). In brief, participants completed at least three acceptable maneuvers, and the highest value was recorded in accordance with standardized guidelines. The 6-MWT was performed to assess submaximal functional capacity following the American Thoracic Society Committee protocol (ATS Committee on Proficiency Standards for Clinical Pulmonary Function Laboratories, 2002).

### Statistical analysis

The primary analysis followed the intention-to-treat principle. Missing post-intervention data were imputed using the last observation carried forward (LOCF) method. Because two participants in the sham group died after baseline assessment and before post-testing, a sensitivity analysis using a complete-case approach (including only participants with post-intervention outcomes) was also performed to evaluate the robustness of the findings. Statistical analyses were performed with the Statistical Package for Social Sciences (SPSS) version 23. Data was verified for normality of distribution using the Shapiro Wilk test. To compare within and between groups (*i.e.,* intervention and control), 2-way mixed ANOVA (Time [2] × Type [2]) was used. The level of significance was set at *p* < 0.05.

## Results

Of the 60 older adults enrolled in the initial study, three participants did not complete the 8-week intervention: one participant in the intervention group attributed his inability to complete the program to a busy lifestyle; the other two participants from the sham-IMT died during the study period due to causes confirmed by the attending physicians as unrelated to the intervention. These events were reported to the institutional ethics committee in accordance with the study protocol. For statistical analyses, these participants were included in the intention-to-treat dataset, with missing post-intervention values imputed using the LOCF method.

Application logs indicated that all participants in both the IMT and sham groups completed all scheduled sessions, corresponding to 100% adherence. No missed or partial sessions were identified, indicating equivalent dose delivery between groups.

Participant safety was monitored throughout the intervention period in accordance with the Declaration of Helsinki and CONSORT 2010 recommendations. At each training or assessment visit, participants were screened for discomfort, adverse symptoms, and changes in health status. Predefined discontinuation criteria (*e.g.*, inability to complete the session due to symptoms) were in place. Any adverse event was immediately documented and reviewed by study clinicians, and serious adverse events were reported to the ethics committee following institutional procedures. Accordingly, 57 older adults were included in the study (intervention group, *n* = 29 and sham group, *n* = 28; Fig. 1). A total of 60 participants were included in the intention-to-treat analysis. For individuals who did not complete the post-intervention assessment, missing outcome data were imputed using the LOCF method. As no intermediate assessments were conducted, baseline values were carried forward in these cases. Two participants in the sham group died after baseline assessment and before post-intervention testing; their baseline values were therefore retained for the primary analysis.

This approach maintains the randomized allocation and preserves sample size while minimizing bias associated with participant dropout. The participants were between 60 and 78 years of age, and one-third were men (23.33%). There were no significant differences in baseline characteristics or cardiorespiratory performance between the intervention and sham-IMT (*p* > 0.05, Table 1). In addition, older adults in both groups showed inspiratory muscle weakness, defined as MIP < 80 cmH_2_O (American Thoracic Society/European Respiratory Society, 2002). Compared to age-specific reference values for healthy adults, participants demonstrated baseline 6-minute walk distances (6-MWD) within the expected range for their age group (Otadi & Malmir, 2026).

**Table 1:** Characteristic data of the participants (*N* = 60).

	Total (*N* = 60)		Breath trainer (*N* = 30)		Sham-IMT (*N* = 30)	
	N (%)	Mean ± SD	N (%)	Mean ± SD	N (%)	Mean ± SD
Sex						
Female (%)	46 (76.67)		26 (56.52)		20 (43.48)	
Male (%)	14 (23.33)		4 (28.57)		10 (71.43)	
Age (yr)		67.68 ± 5.45		66.67 ± 4.84		68.70 ± 5.91
BMI (kg/m^2^)		26.18 ± 3.85		25.88 ± 3.95		26.49 ± 3.80
MIP (cmH_2_O)		65.65 ± 15.63		64.20 ± 18.91		67.10 ± 11.63
MEP (cmH_2_O)		65.18 ± 16.97		61.90 ± 18.85		68.47 ± 14.43
6MWT (meters)		404.48 ± 83.01		409.40 ± 90.83		399.57 ± 75.62

**Notes.**

SD standard deviation BMI body mass index MIP maximal inspiratory pressure MEP maximal expiratory pressure 6MWT 6-minute walk test

For comparison of respiratory muscle strength and submaximal functional capacity between intervention and sham-IMT, two-way mixed repeated ANOVA was conducted to compare differences within and between intervention and sham-IMT (Table 2). After completing the 8-week intervention program, older adults who used Breath Trainer showed high MIP (Δ23.37 ± 2.52 cmH_2_O, 95% CI [18.33–28.41], n^2^_p_ = 0.597, *p* < 0.001; the sham-IMT showed no significant increase (Δ0.90 ± 2.52 cmH_2_O, 95% CI [−4.14 to −5.94], n^2^_p_ = 0.002, *p* = .722). In addition, the intervention group, but not the sham-IMT, displayed increased MEP (Δ25.43 ± 2.55 cmH_2_O, 95% CI [20.32–30.54], n_p_^2^ = 0.631, *p* < .001). Regarding submaximal functional capacity (defined as 6-MWT), the intervention group showed improved 6-MWD (Δ28.87 ± 5.81 m, 95% CI [17.23–40.50], n^2^_p_ = 0.298, *p* < .001), whereas the sham-IMT showed a significant decrease (Δ −17.93 ± 5.81 m, 95% CI [29.57–6.30], n^2^_p_ = 0.141, *p* = .003).

**Table 2:** Comparison between cardio-respiratory performance breathing training and sham-IMT in older people after 8-week intervention program.

	Breath trainer		Change (mean ± SE)	Sham-IMT		Change (mean ± SE)	*p*-value breath trainer *vs.* Sham-IMT
	Pre-test	Post-test		Pre-test	Post-test		
MIP (cmH_2_O)	64.20 ± 2.87	87.57 ± 3.21	23.37 ± 2.52^***^	67.10 ± 2.87	68.00 ± 3.21	0.90 ± 2.52	<0.001
MEP (cmH_2_O)	61.90 ± 18.85	87.33 ± 11.32	25.43 ± 2.55^***^	68.47 ± 14.43	68.47 ± 14.82	0.00 ± 2.55	<0.001
6MWT (meter)	409.40 ± 90.83	438.27 ± 78.47	28.87 ± 5.81^***^	399.57 ± 75.62	381.63 ± 59.40	−17.93 ± 5.81^**^	0.003

**Notes.**

** *p* < 0.01.

*** *p* < 0.001.

SE standard error MIP maximal inspiratory pressure MEP maximal expiratory pressure 6MWT 6-minute walk test

The improvement observed in the IMT group exceeded the commonly reported minimum clinically important difference of 20–40 m for older adults, indicating a clinically meaningful change (Perera et al., 2006; Daynes et al., 2025).

Additionally, comparing between-group, the intervention group had higher MIP; Δ 19.56 ± 4.54 cmH_2_O, F (1, 58) =18.55, n^2^p = 0.242, *p* < 0.001, MEP; Δ 18.87 ± 3.41 cmH_2_O, F (1,58) = 30.71, n^2^p = 0.346, *p* < 0.001, and submaximal functional capacity values; Δ 56.63 ± 17.97 meters, F (1, 58) = 9.93, n^2^p = 0.146, *p* = 0.003 than the sham-IMT group.

In addition, a complete case-sensitivity analysis excluding participants without post-intervention data was performed. The results were consistent with the intention-to-treat LOCF analysis. Between-group differences in the primary outcomes remained statistically significant, and the magnitude and direction of the effects were comparable (Table S1).

## Discussion

We developed a novel IMT device, the Breath Trainer, and explored the effects of IMT on cardiorespiratory performance in older adults. We hypothesized that inspiratory muscle training would lead to significant improvements in respiratory muscle strength and submaximal functional capacity compared with a sham condition in older adults with low physical activity. During the 8-week intervention program, 30 participants were instructed to attach the developed device with 40% MIP, while another 30 used the Breath Trainer with 0% MIP. The results revealed that the intervention group had increased respiratory muscle strength and submaximal functional capacity compared with the sham-IMT.

IMT has been shown to improve respiratory muscle strength and submaximal functional capacity in older adults, those with chronic illness, and those with cardiovascular disease (Beaujolin et al., 2024; Katayıfçıet al., 2024). A meta-analysis of seven studies involving 248 participants revealed that IMT could improve inspiratory muscle strength and diaphragmatic muscle thickness upon training at an intensity of 30–80% MIP, 5–7 sets per week, and a total training time of 4–8 weeks (Seixas et al., 2020). A systematic review of 13 studies reported that 4–8 weeks of IMT (50–70% of MIP, 7 days/week) could enhance cardiac autonomic and vascular functions (Mello et al., 2024). Inspiratory muscle weakness is defined as maximal inspiratory muscle pressure of ≤ 70 cmH_2_O in women and ≤ 80 cmH_2_O in men (American Thoracic Society/European Respiratory Society, 2002; Laveneziana et al., 2019). In the present study, 13 of 29 participants who underwent IMT showed reversibility from inspiratory muscle weakness to normal inspiratory muscle weakness, whereas reversibility was observed only in two participants in the sham-IMT (two of 28 participants). IMT is associated with increased MIP, which contributes to improved diaphragmatic thickness and increased muscular hypertrophy (Souza et al., 2014; Seixas et al., 2020; Enright et al., 2006; Mills et al., 2015). Moreover, IMT reportedly attenuates the reduced oxygen saturation in intercostal muscles during respiratory fatigue in patients with chronic heart failure (Moreno et al., 2017). Inspiratory training can increase intercostal internal muscles’ strength, generally considered muscles of expiration (Sasaki, Kurosawa & Kohzuki, 2005). Therefore, using Breath Trainer could enhance inspiratory and expiratory muscle strength.

Increased respiratory muscle strength has been found to decrease diaphragmatic fatigue; in other words, it can improve diaphragmatic hypertrophy and enhance ventilation efficacy. Additionally, IMT reduces muscle metabolites during exercise, resulting in attenuated vasoconstriction responses in the lower limb (Chiappa et al., 2008; Plentz et al., 2012). Therefore, IMT may increase submaximal functional capacity in older adults (Elabd et al., 2024). Following IMT, three studies assessing 101 older adults reported an average 6-MWD of 24.7 ± 22.1 m (Manifield et al., 2021). In the present study, 6-MWD was increased to 29.86 ± 5.99 m. This improvement suggests enhanced functional exercise capacity following inspiratory muscle training. Previous studies have proposed that such improvements may be related to modulation of the respiratory muscle metaboreflex, a sympathetic nervous system–mediated response during exercise that can reduce blood flow to locomotor muscles through vasoconstriction (Moreno et al., 2017; Chiappa et al., 2008; Witt et al., 2007). It has been suggested that improved oxidative capacity of the inspiratory muscles may attenuate sympathetic activation and thereby support oxygen delivery to peripheral muscles during exercise (Moreno et al., 2017; Chiappa et al., 2008; Archiza et al., 2018). However, these physiological variables were not directly measured in the present study. Therefore, our interpretation focuses on the observed improvements in respiratory muscle strength and functional capacity. Nonetheless, the proposed mechanisms remain hypothetical and should be explored in future investigations. Respiratory threshold training commonly involves breathing through the mouth, which may utilize an accessory respiratory muscle. However, the Breath Trainer could focus on the major inspiratory muscles (*i.e.,* the diaphragm and intercostal muscles). As Breath Trainer must be attached to the lower margin of the chest wall, it provides an inspiratory threshold loading focused on the inspiratory muscles, particularly the lower costal and diaphragmatic muscles. The protocol included an initial load of 40% MIP, performed 15 times/set, three sets/day, for 5 days/week.

The external abdominal cuff that we used increases diaphragmatic load by mechanically opposing abdominal expansion, a mechanism distinct from mouth-applied resistive or threshold devices that impose an intraluminal airway load. If participants adopt thoracic (upper-chest) breathing during training, then the cuff may not substantially increase diaphragmatic work, and observed benefits could partly reflect respiratory-pattern exercise, improved breathing coordination, or general physical activity rather than isolated inspiratory muscle overload. We mitigated this risk by providing standardized diaphragmatic breathing instruction and supervised practice before home training. Nevertheless, as we did not directly measure diaphragm activation (*e.g.*, ultrasound and electromyography) or breathing pattern during home sessions, we cannot exclude that some portion of the effect arose from respiratory exercise or improved motor control rather than pure strength training of the inspiratory muscles. The larger increases in MIP observed in the intervention group (relative to sham) are consistent with inspiratory muscle adaptation, but future studies should include objective measures of diaphragmatic recruitment to confirm the mechanism.

The study does have several limitations. Almost 75% of the participants were women, which might have affected the representation of the overall older population. Future studies should recruit a more balanced sample or examine whether sex-specific differences influence responsiveness to IMT. Participants were selected based on the criterion of GPAQ < 600 MET-min/week physical activity level, but variables such as actual physical fitness (maximum oxygen uptake and muscle strength) were not controlled. Further, two deaths occurred in the sham group during the study period. Both events were reviewed by medical staff and confirmed to be unrelated to the intervention, and they were reported promptly to the ethics committee. These participants were included in the intention-to-treat analysis using the LOCF method; however, the loss of post-intervention data may influence interpretation of group differences. Simple measurements, *e.g.*, 6-MWT were used in the assessment. However, more sophisticated measurements, such as measuring the maximum oxygen uptake, heart rate variability, and pulmonary function tests to estimate cardiorespiratory performance, or using ultrasound to measure diaphragm thickness, could provide more accurate evaluation of physiological changes. Therefore, comprehensive cardiorespiratory assessments may provide deeper insight into the physiological mechanisms underlying IMT responsiveness. Additionally, resistance in this trial was applied externally through an abdominal cuff, and we did not directly quantify whether participants consistently used diaphragmatic breathing during unsupervised home sessions. Consequently, the observed improvements may result from a combination of mechanisms, inspiratory muscle strength gains, improved breathing pattern/coordination, and general respiratory exercise rather than isolated diaphragm hypertrophy. Future research should (1) incorporate objective measures of diaphragmatic function and breathing pattern during training (*e.g.*, diaphragm ultrasound to assess thickening fraction, respiratory inductance plethysmography to quantify thoraco-abdominal movement, or surface/intramuscular electromyography), (2) compare abdominal-cuff and mouth-resistive methods directly, and (3) evaluate different training intensities to determine dose–response relationships in older adults.

## Conclusions

Collectively, the findings of this study revealed that novel IMT with a smartphone-linked device produced considerable improvements in respiratory muscle strength and functional capacity in older adults with low physical activity levels. The present findings support the potential role of smartphone-assisted inspiratory muscle training as a feasible component of community-based rehabilitation for older adults that emphasizes prevention of functional decline for older adults. However, the results should be interpreted cautiously given the study’s sample characteristics and methodological limitations. Future work should include more diverse participants and incorporate comprehensive physiological assessments to further clarify the mechanisms underlying IMT-related.

## Supplemental Information

10.7717/peerj.21072/supp-1 Supplemental Information 1 Raw data

10.7717/peerj.21072/supp-2 Supplemental Information 2 Comparison between cardio-respiratory performance breathing training and sham-IMT in older people after 8-week intervention program (n = 57)

10.7717/peerj.21072/supp-3 Supplemental Information 3 CONSORT checklist

## Additional Information and Declarations

### Data Availability

The following information was supplied regarding data availability: The raw data is available in the Supplemental File.

### Clinical Trial Registration

The following information was supplied regarding Clinical Trial registration: TCTR20211006002.
