# Supplementary material for: Beneficial effects of a novel inspiratory muscle training device on respiratory muscle strength and submaximal functional capacity in community-dwelling older Thai adults: a randomized controlled trial
Source: PeerJ. 2026 Apr 7;14:e21072. doi: 10.7717/peerj.21072 (PMC13068012; doi:10.7717/peerj.21072)
Supplement: Supplemental Information 2 [file peerj-14-21072-s002.docx]

Supplementary Table S1: Comparison between cardio-respiratory performance breathing training and sham-IMT in older people after 8-week intervention program (n= 57)

|  | Breath Trainer (n=29) | | Change  (mean ± SE) | Sham-IMT (n=28) | | Change  (mean ± SE) | p-value  Breath Trainer vs.  Sham-IMT |
| --- | --- | --- | --- | --- | --- | --- | --- |
|  | Pre-test | Post-test |  | Pre-test | Post-test |  |  |
| MIP (cmH_2_O) | 64.72±190.2 | 88.90±16.00 | 24.17±2.56*** | 67.04±11.80 | 68.00±18.34 | 0.96±2.61 | <.001 |
| MEP (cmH_2_O) | 61.17±18.75 | 87.48±11.49 | 26.31±2.59*** | 68.00±14.64 | 68.00±15.05 | 0.00±2.63 | <.001 |
| 6MWT (meter) | 407.55±91.86 | 437.41±79.72 | 29.86±5.99*** | 402.57±76.97 | 383.36±60.54** | -19.21±6.10 | 0.006 |

^**^*p*<.01, ^***^ *p*< .001, SE: standard error, MIP: maximal inspiratory pressure, MEP: maximal expiratory pressure, 6MWT: 6-minute walk test
